# Round the Hospitals

**Published:** 1919-08-23

**Authors:** 


### ROUND THE HOSPITALS.

THE movement in favour of granting the war medal for home service has its roots in a desire for fair play, but there can be no doubt that, were it successful, it would enormously depreciate the value of the medal in the eyes of its recipients. A writer in the *Times* urges that the home service nurses are entitled to this distinction on the grounds that the vast majority of those who stayed at home had no choice and were deeply disappointed, and that home service was "the more arduous of the two." It is slightly ridiculous to talk of dimming the honour of the award "with such obvious and far-reaching injustice," After all, the war medal is intended for nurses who were at the war. To extend it to those who stayed at home would be to alter its character fundamentally. We yield to none in honour for the single-minded matrons and sisters of military and general hospitals who carried on at home. And what about the district nurses, who hoed a lonely and hard furrow during the same period? What about the countless labourers in Poor-Law infirmaries, sick wards, fever

**ROUND THE HOSPITALS**—(*continued*).

hospitals, institutions for the incurable, and the like? Would not most of these also have willingly exchanged their lot for that of the war nurses? Let us honour whole-heartedly all who stayed behind. But that is a very different thing from grudging a little special distinction to those who upheld the honour of the profession abroad.

---

THE new assistant inspectorships in the Insurance department under the Ministry of Health offer uncommon prospects to trained nurses with the requisite qualifications and of good previous education. We trust that among the candidates who applied before the appointed day (August 15) were many of the demobilised sisters, and that in subsequent appointments women trained in hospitals will be able to prove their fitness for this branch of the Civil Service. The posts start at £100 plus £40 war bonus and rise by annual increment of £10 to £300. Superior posts rise to £400. Thirty-six days annual leave is allowed. Superannuation allowance is made on retirement, which is compulsory at 60. So far there is no competitive examinaton for these posts, merely a qualifying examination for such as do not possess a university degree. No doubt as time goes on more precise regulations will be laid down for the training of these inspectors on whom the good administration of the National Insurance (Health) Acts will largely depend. And it may be permitted to hope that a period of institution training will be recognised as one of the portals to the service. We do not know any other form of training which so surely inculcates an adequate sense of the importance of detail in regard to matters of health.

---

BOTH the *Times* and the *Daily Telegraph* have sympathetic articles on the nursing sisters who are out of work after fine exertions during the war. In their zeal to suggest openings for these ladies, whose qualifications are, for the most part, quite above the average, the lay-papers are not always in accord with knowledge. For instance, the *Times* suggests that the sisters should be invited to become stewardesses on board ships and matrons in boys' and girls' schools. It is true some young nurses with a restless desire for travel are always besieging the shipping offices for a chance of going to sea. But the duties of a stewardess, her standing and unfortunate dependence on tips, are not in keeping with the status of the trained nurse. And although a few of the higher-class schools do undoubtedly offer a suitable stipend and position to the sanatorium sister, school matronships, as a rule, are almost purely domestic in character. We are disposed to think that the ranks of private nursing are still understocked and that quite a couple of hundred nursing institutes in London and the Provinces might enlarge their staffs, and could do so without risk, in anticipation of the autumn flow of business.

---

A LARGE number of names is published this week which have been brought to the notice of the Secretary of State for War by the Chairman of the Joint War Committee of the British Red Cross Society and Order of St. John of Jerusalem in England for valuable nursing services rendered in connection with the war. The lists are arranged territorially, and contain the names of lady superintendents, sisters-in-charge, head sisters, staff nurses, and V.A.D. nurses in Anglesey, Bedfordshire, Berkshire, Cambridgeshire, Cardiganshire, Cheshire, Cornwall, Cumberland, Denbighshire, Derbyshire, Devonshire, Dorsetshire, Durham, Essex, Glamorganshire, Gloucestershire, Hampshire, Herefordshire, Hertfordshire, Kent, Lancashire, Leicestershire, Lincolnshire, London, Merionethshire, Middlesex, Monmouthshire, Montgomeryshire, Norfolk, Northumberland, Nottinghamshire, Oxfordshire, Radnorshire, Rutland, Shropshire, Somersetshire, Staffordshire, Suffolk, Surrey, Warwickshire, Westmorland, Wiltshire, Worcestershire, Yorkshire. We see no mention of Sussex. There are also many names in Scotland and Ireland.

---

A FURTHER list was issued on August 19, of ladies who have been brought to the notice of the Secretary of State for War, for valuable nursing services rendered in connection with the war. They comprise sisters, staff-nurses and V.A.D. nurses in the Aldershot Command, the Eastern Command, the London District, the Northern Command, the Scottish Command, the Southern Command, the Western Command, the Dominion of Canada, the Commonwealth of Australia, the Dominion of New Zealand, the Union of South Africa, and some from Malta and Gibraltar. A large number of the sisters belong to Q.A.I.M.N.S.R. and the T.F.N.S. while some are civil hospital sisters who had charge of wards for the wounded.

---

NURSING sisters are to take a prominent place in the pictorial representation of war activities now in course of preparation for the Imperial War Museum. We understand that Mr. William Nicholson is engaged on a picture of Army Nurses in the Peace Procession, at the moment when they saluted the Cenotaph. Mr. George Harcourt has chosen for his brush the march of the V.A.D. nurses in the Procession as they passed the King, with Lady Ampthill at their head. Sir John Lavery is engaged on studies of the Army Sisters and Red Cross workers in their duties in France. The winter exhibition of the Woman's Section at Burlington House will be found to be full of interest for war workers, and the Devonshire House ballroom full of V.A.D. workers by Miss Claire Attwood will be a popular picture.

---

THE asylum staffs of the Metropolitan Asylums Board through their representatives have agreed to a working week of sixty hours, inclusive of mealtimes, which are reckoned at ten hours a week. This makes a fifty hours' week which carries fourteen days' leave every six months. The staff unanimously preferred this to the alternative arrangement of a forty-eight hours' week and fourteen days' leave once a year. Most people indeed

**ROUND THE HOSPITALS—(*continued*).**

would prefer to put in an extra couple of hours work a week if thereby they could earn an extra fortnight's holiday and there is little doubt the nurses in the M.A.B. institutions will declare in favour of this plan in their approaching ballot. The Report on the hours of duty and payment of institution staffs prepared by the Committee of the Board is likely to reckon as a Magna Charta in institution life. It sweeps away many crusted abuses, and aims at making service under the M.A.B. a coveted distinction.

One of the most interesting features of the reforms proposed by the M.A.B. is the introduction of over-time payments for all members of the staffs below the rank of assistant matron or head attendant. It will be calculated at the rate of time and a quarter for the first and second hours, and thereafter at the rate of time and a half, on a weekly basis. We have always been convinced that overtime must come if the limitation of working hours was to be a reality. The needs of the sick are incalculable, and in practice, however carefully the probationers' interests are safeguarded, extra work must fall on them occasionally. Overtime pay is the best protection they can have, because when it mounts up, it betrays the fact that insufficient provision has been made for the incidence of emergencies, and being an expensive expedient, it will ensure the engagement of emergency nurses.

Probably more nurses are taking their hard-earned holiday at this moment than ever before in the history of nursing. Many of these peace holiday-makers are idle perforce. Many, we fear, are discovering that holidays are a very expensive luxury. But one and all are storing up fresh energy for the winter campaign and are thoroughly happy, if not care-less, especially those whose lot it has been to forgo most of their holidays during the war. One interesting discovery has been made by some of these self-sacrificing people who went on working while others rested, and that was that a fortnight's rest was quite enough to send them back to work with renewed zest and that a month of idleness was positively too much, veering round to boring point. There is a great deal to be said in favour of the double holiday, fourteen days twice a year, as instituted by the M.A.B. If it could only be combined with the overseas arrangement, whereby heads of institutions and departments after a term of years get six months' holiday at full pay, the lot of nursing sisters would be a widely different one, and nerve-strain a thing of the past.

The Ministry of Health has promptly taken up the cause of the blind, which up to the present time has been left to the unaided resources of certain admirable societies, many of which are near to breaking-point from the intolerable strain. After consultation with societies most nearly concerned, a set of regulations has been issued under which, subject to the consent of Parliament, grants will be made for services rendered to the blind by philanthropic agencies. The maintenance of workshops for the blind, of homes and hostels, of teaching and training centres are among the enterprises which will now be subsidised, and managers should at once procure a set of the regulations (price 3d.) from the Stationers' Office or through a bookseller. Proposals are to be laid before Parliament without delay for dealing with the unemployable blind. The extension to this class of sufferer of the equivalent of the old-age pension has long been over-due, and they ought wherever possible to be taken from workhouse wards. A vigorous protest on their behalf took place in Trafalgar Square last Sunday.

The Lord Mayor is appealing for further donations on behalf of the Children's Country Holiday Fund, with a view to extending the benefit of a fortnight in the country to 20,000 London children this year. We would on no account put a spoke in his wheel, but we would earnestly urge caution on those who send children down to overcrowded country villages this summer. Unless experienced visitors in each locality can arrange for the children having adequate accommodation, it is far better to leave them for once in London. The overcrowding in many of the places where Londoners used to be received makes it most difficult, even at greatly increased rates, to get children taken in good homes, and bad country homes, dirty, airless, rough, and low in tone, benefit none. The real remedy for the impasse seems to be in the establishment of a dozen or more open-air camps under the auspices of the Children's Country Holiday Fund, where good food, exercise, games, and baths could be enjoyed by the little Londoners under the most favourable conditions, with a little good influence thrown in. Perhaps this may be done another year.

Many nurses may find themselves affected by the new National Health Insurance Act, which has just received the Royal assent. The limit of remuneration within which persons employed, otherwise than by way of manual labour, are liable to compulsory health insurance has been raised from £160 to £250 per year. Certificates of exemption from their own payments, but not from the employer's contribution, can be obtained under certain conditions, and forms of application for such exemption can be obtained at any post office. The new provisions will affect the clerical staff in hospitals, the matron, and other officials whose income has hitherto placed them outside the necessity for health insurance.

Great regret will be felt among the staff of Lady Minto's Nursing Association in India at the news of Dame Sidney Brown's resignation from the office of secretary. A large share of the success which has attended the work of the Association is undoubtedly due to the admirable management at the home headquarters, where promptness and business ability have been allied to very kindly sympathy and an almost uncanny talent for choosing the right people. The new secretary is Miss Ray, R.R.C., 54 Ashburnham Mansions, Chelsea, and everyone

**ROUND THE HOSPITALS**—(*continued*).

knows that the affairs of the Association will be very safe in her hands. There will be vacancies to be filled in the autumn, and early application should be made to Miss Ray at the above address. The certificate of the C.M.B. is a *sine qua non*. Salaries commence at £90 and rise to £135, with everything provided.

In issuing the 170th Report of the Liverpool Royal Infirmary, the authorities are able to make the announcement that a 56-hour week for nurses and probationers has been working smoothly since June 1. The sisters work 47 hours. The Committee have decided that it is not an opportune moment to appeal to the public for the very large sum needed for a new Nurses' Home, but are taking immediate steps to secure better accommodation for those nurses who cannot be housed in the present home. The matron reports that the difficulty of carrying on was greater than ever last year. The number of candidates sank from 1,600 in the years preceding the war to 570, the majority far below the usual standard. Nine sisters left during the year, mostly to take higher posts elsewhere. A total of 84 sisters and nurses have left the Royal Infirmary staff to do naval or military work since war began. Four more sisters gained the R.R.C. during the year; Miss Hutton, first class; the Misses Swift, Hunt and Garner, second class. Miss Rosetta Miller has gained the R.R.C., has been twice "mentioned," and has received the Médaille des Epidémies in addition to the Mons Star. The Misses Garner and Elsie Johnson also have the Mons Star. The new massage department now consists of a sister-in-charge, 1 resident masseuse and 9 non-resident; 14 pupils were prepared for the I.S.T.M. certificate.

The important decision arrived at by the Council of the Incorporated Society of Trained Masseuses in lengthening the prescribed course of training for entrants to their examinations to one year of forty-eight weeks, cannot fail to have the effect of appreciably raising the standard of British Schools of Massage and remedial exercises. From September 1, no new schools are to be accepted by the Council unless a year's training be provided, either in massage only or in massage and Swedish exercises conjointly, and no new teacher of massage will be accepted after July 1, 1919, unless he or she hold the Society's certificate in Swedish remedial exercises or other approved certificate, as well as the massage certificate. The date after which examinees must produce evidence of having received one year's training is January 1, 1921, so that next year's candidates are not affected. There is abundant evidence to show that these subjects cannot be properly mastered in less than a year, and some of the schools which maintain an eighteen-month or a two-year course, are overcrowded with applicants.

Nurse Raine has gained the Frater gold medal at the Tynemouth Poor Law Hospital, Nurse Cowell the Murray medal, and Nurse Burton the Pearson medal. The examiner was Dr. Parker, and all the third-year probationers obtained their certificate. The medical officer reports that the sequence of probationers has been broken, owing to many having left before completing their training, and that by May in next year, unless steps are taken, there will be nineteen first-year probationers out of a staff of twenty-eight, instead of only nine or ten. The dilemma is not an infrequent one, but, unhappily, is not always followed by prompt measures. In the present instance the Chairman of the hospital Committee, the medical officer, and the superintendent nurse have been charged with the duty of preparing a scheme for putting matters right.

Congratulations to the nursing staff of the Bermondsey Infirmary on the adoption by the Guardians of a forty-eight-hour week for all their employees. This means the addition of a home sister and twelve extra nurses, and we understand the matron is rather perplexed how to find room for them.

The Cheshire County Nursing Association trains five nurses every year, at a cost last year of £376, which was defrayed by the County Council. The annual meeting of the Association was held at Crewe under the chairmanship of Colonel Sir George Dixon, who spoke vigorously of the miserable homes in many parts of the county, the shocking overcrowding, and the absence of conveniences essential to health. Salaries of district and village nurses are being raised in accordance with the recommendations of the Queen Victoria Jubilee Institute. The new County Superintendent appears to be doing excellent work, three new districts have lately affiliated and the affairs of the Associated are being managed with ability. But an enormous expansion is needed if the county of Cheshire is to be adequately provided with trained nurses and for this increased financial support is imperatively called for.

During the next six weeks the marrow will be in full season, and it is really too precious to be wasted, as it frequently is in institutions, by being served as a watery and unappetising dish, partly hidden, maybe, by sticky flour-and-water paste. The housekeeper should lay hands on all that she can procure, and, having peeled and seeded them, should boil them down for pulp. In pre-war days an equal weight of sugar was allowed, but half this must suffice nowadays. A cupful of water should be allowed to each pound of marrow, and the sugar and water boiled to a clear syrup. Add the marrow and simmer for an hour and a half, until all is thick and transparent, taking care it does not burn. If marmalade is required, the rind of half a lemon, chopped small, must be allowed to every pound of marrow and must be added to the mixture at the end, all being boiled together for a quarter of an hour. If preserve is wanted, allow the rind and juice of a lemon and half an ounce of ginger to each pound of marrow. Almost any kind of fruit—plum, blackberry, damson, apricot—may be used with the marrow in any proportion desired, half and half, and will convey its distinctive flavouring to the whole.

